# Guided self-help for anxiety among Huntington’s disease gene expansion carriers (GUIDE-HD) compared to treatment as usual: a randomised controlled feasibility trial

**DOI:** 10.1186/s40814-023-01364-5

**Published:** 2023-09-12

**Authors:** Maria Dale, Fiona J. R. Eccles, Katie Melvin, Zaynah Khan, Lee Jones, Nicolò Zarotti, Reza Kiani, Jenny Johnson, Robert Wells, Jane Simpson

**Affiliations:** 1 Leicestershire Partnership NHS Trust, Mill Lodge, Narborough, Leicestershire, LE19 4SL Leicester UK; 2 Division of Health Research, Faculty of Health and Medicine, Lancaster University, Lancaster, LA1 4AH UK; 3 Manchester Centre for Clinical Neurosciences, Salford Royal Hospital, Salford, M6 8HD UK; 4 Leicester, UK

**Keywords:** Feasibility, Psychological intervention, Guided self-help, Huntington’s disease, Anxiety

## Abstract

**Background:**

Huntington’s disease (HD) is an adult-onset genetic neurodegenerative condition associated with cognitive decline, motor impairments, and emotional difficulties. Anxiety affects up to 71% of HD gene expansion carriers (i.e., those with the version of the gene that causes HD) and can negatively impact quality of life, worsen other HD symptoms, and increase suicide risk. Therefore, helping people with their anxiety should be a clinical priority. A significant evidence base now exists for low-cost talking therapies for anxiety, such as guided self-help, and with people with other neurodegenerative conditions (e.g., Parkinson’s disease). However, this type of intervention has not been specifically assessed with HD gene expansion carriers.

**Methods:**

This protocol describes an exploratory randomised controlled feasibility study of a psychological intervention for anxiety for HD gene expansion carriers. The 10 session guided self-help intervention ('GUIDE-HD') is based on a blend of second and third wave cognitive behavioural models of anxiety (cognitive behaviour therapy [CBT] and acceptance and commitment therapy [ACT]) and is adapted to meet the specific needs of an HD population. This study will compare guided self-help with treatment as usual (TAU), with 15 HD gene expansion carriers randomly allocated to each group. Participants will be recruited across the UK. Quantitative data will be collected pre-intervention, immediately post-intervention, 3-month post-intervention and 6-month post-intervention. Qualitative data will be collected at one month post-intervention from participants, including HD carers. The data will be analysed to assess whether the current intervention and study design are feasible to progress to a larger randomised controlled trial. Feasibility has been defined in terms of recruitment rate, retention rate to both trial arms, intervention adherence, and acceptability of the intervention and measurement tools.

**Discussion:**

Given the lack of evidenced interventions to date to support the wellbeing of people with the expanded Huntington’s gene, this study will assess the feasibility of progressing this particular intervention to a full trial. To try and increase the acceptability of the intervention, a number of stakeholders, including those affected by HD and in caring roles, have been fundamental to the creation of the intervention (e.g., therapy manual, planned therapy process) to date.

**Trial registration:**

Trial ID: ISRCTN47330596.

Date registered: 28/09/2022.

Protocol version and date: Version 2, 09/06/22.

Trial sponsor organisation and contact: Leicestershire Partnership NHS Trust (Dave Clarke).

Role of sponsor: Overall responsibility for the conduct and governance of the trial.

Role of funder: Review of initial research proposal.

## Background

Huntington’s disease (HD) is a genetic neurodegenerative condition caused by the expansion of a gene (HTT) on a chromosome linked to basal ganglia function, and particularly the corpus striatum [1]. Once individuals with the gene expansion are symptomatic - often in their 30s-40s, although juvenile onset is possible [2] - increasing damage to these neurological areas progressively affects a number of functions associated with movement and cognition [3]. Individuals also often experience a range of psychological difficulties, although the onset/development of these is not uniquely linked to neurodegeneration [4]. For example, they can occur before the onset of movement difficulties [5] and be related to the difficulties and challenges encountered in growing up in a family where perhaps a number of relatives (including a parent) have HD [6]. As with many neurodegenerative conditions [7], psychological difficulties can also emerge through the experience of living with the challenges and uncertainties of a chronic health condition in a society where stigmatising attitudes to illness are still evident (e.g., [8, 9]).

One difficulty which is well documented in people affected by Huntington’s disease is anxiety, experienced by up to 71% of individuals with the gene expansion and negatively affecting every-day functioning, independence and quality of life [10]. Moreover, people with the HD gene expansion can experience emotional difficulties approximately 15 years before motor signs [11] and are equally affected by anxiety as those with the clinical diagnosis [10]. HD gene expansion carriers with anxiety also report greater depression, irritability, apathy, pain, involuntary movements/chorea, and suicidality [10, 12–16]. Higher anxiety has also been found to predict greater workplace impairment for premanifest (i.e., not motor symptomatic) HD gene expansion carriers [17]. Therefore, reducing anxiety has the potential to improve a number of outcomes.

The current evidence around the effectiveness of targeted psychological interventions for people with HD (pwHD) and their effectiveness for different psychological outcomes is limited [18]. Indeed, there is an absence of any research on psychological interventions specifically targeted at anxiety in people at any stage of HD. Nonetheless, recent expert guidelines have recommended psychological therapy as the first treatment offered to people experiencing anxiety in early-stage HD [19].

Guided self-help is a cost-effective psychological therapy for anxiety and involves support from a psychological therapist to ‘guide’ the patient to use a self-help intervention [20]. Guided self-help predominantly uses techniques from cognitive-behavioural therapies (CBTs), evidence-based psychological interventions which have shown promising results in other similar conditions such as multiple sclerosis and Parkinson’s disease [21–23]. In a systematic review of anxiety among HD gene expansion carriers, it was concluded that anxiety was associated with several modifiable targets of change used in evidence-based psychological treatments, such as CBT [10]. Another recent study found that people with HD who used greater coping strategies involving adapting to the stress of having HD experienced fewer symptoms of anxiety [24]. These coping strategies include acceptance, changing unhelpful appraisals, and undertaking positive activities - all of which are included in cognitive behaviour therapies such as traditional second-wave CBT and third wave therapies, such as Acceptance and Commitment Therapy (ACT). Moreover, pwHD are accepting and open to psychological interventions [25].

Consequently, we present here a protocol of an exploratory randomised controlled trial (RCT) to assess the feasibility of conducting a full RCT on the effectiveness of a guided self-help CBT-based intervention (i.e., a blend of CBT and ACT) for anxiety in both people with premanifest and early HD (GUIDE-HD). Early-stage HD is being targeted as this psychological approach is considered unsuitable for those with significant cognitive and/or communication difficulties.

## Methods/design

### Aims

The overall aim of this exploratory trial is to test the feasibility of an RCT evaluating the clinical effectiveness of a psychological intervention, compared to usual care, in reducing anxiety in individuals with pre-manifest and early-stage HD.

Specific objectives for the study are as follows:

- Assess the feasibility of recruitment and retention to both arms of the trial (intervention versus treatment as usual) across assessment, intervention, and follow-up periods of three and six months (primary objective).
- Describe participant characteristics and assess generalisability compared to HD populations more widely.
- Describe screening assessment results.
- Assess the feasibility of measuring the primary and secondary outcomes and obtain information to inform the sample size for a full RCT.
- Describe and explain the fidelity to the intervention and evaluate views, experiences, and acceptability of the participants.
- Investigate the acceptability of the intervention (including to carers) and outcome measures.
- Determine whether criteria to progress to a definitive trial are reached.

### Intervention: rationale and development

The guided self-help intervention in the treatment arm ('GUIDE-HD') is based on cognitive-behavioural models of anxiety and is being adapted to meet the specific needs of an HD population. The intervention uses both content and process-based cognitive behaviour therapies, using a recent framework [26] whereby evidence-based processes of change will be targeted. The approach is a blend of traditional CBT and ACT for anxiety, including psychoeducation around anxiety, identifying one’s values, relaxation, exposure, acceptance, managing thoughts (e.g., cognitive defusion techniques and identifying cognitive biases) and problem-solving. During the intervention, participants will be provided with a toolkit of resources containing psychological techniques that are known to help reduce anxiety and designed specifically for HD. Building upon guided self-help CBT practices in other neurological conditions [21, 22], HD-specific guided self-help workbooks and a therapist manual were developed. The intervention focus was borne through clinical experience of unavailable standardised psychological interventions adapted for people affected by HD. A number of stakeholders were involved in the design and generation of materials for the intervention. The initial design for the intervention arose from feedback from both local and national UK patient and public involvement (PPI) groups of HD family members, whereby the importance of HD-specific examples and carer involvement in the intervention was highlighted. The workbooks and manual were written by clinical psychologists with a combined 15 years of psychology practice in HD services, with additional feedback from HD clinical academic psychologists and other HD clinicians (e.g., nursing staff) and co-produced with a steering committee of people personally affected by HD and anxiety. A series of face-to-face meetings and email correspondence with the HD steering group totalled approximately 12 hours over nine months to review initial drafts, revise drafts, and produce the final materials. Feedback was welcomed on the planned intervention alongside the content and format of the corresponding materials. All recommendations made were accepted; specific examples for the intervention workbooks included adjusting the gender ratio of illustrations, adding page numbers, reducing references to family while addressing intergenerational experiences of HD, adding examples of HD-relevant coping strategies (e.g., avoiding reminders of HD, hypervigilance of potential signs or changes in HD symptoms), reordering the content of materials, and simplifying the visual design templates. Broader recommendations discussed by the steering committee included the value of diverse illustrations (e.g., different ethnicity, age, ability representations), the acknowledgment of challenging situations and dilemmas pwHD encounter as they navigate their relationships and circumstances, the understandable distress and coping strategies that can arise, and the sensitivity of topics which the steering committee found most emotive (namely acceptance and the relationship between HD and anxiety). High-quality colour workbooks were key for this guided self-help intervention.

This guided self-help intervention follows a weekly cycle, starting with the participant being given a workbook to review in their own time at their own pace and followed by a facilitated session to review and support their understanding. The workbooks involve reflective exercises for participants to write about how the material relates to them or suggestions of activities to try. Based on individual preferences, participants will receive either a weekly telephone, video call, or email-guided psychological support across 10 sessions (aimed at weekly sessions lasting one hour). Adaptations to standard CBT will be made to compensate for mild cognitive impairments, including the use of external memory aids and behavioural routines. Where applicable, carers/partners will be offered copies of the toolkits to support the participant as well as a maximum of three individual sessions with the facilitators across the intervention period, to help them support the pwHD.

As a structured intervention, adherence to the intervention is planned through supporting the participants and facilitators. Participant adherence to the intervention is supported through integrating behaviour change considerations into the sessions. In workbook 1, participants will identify how their engagement may be impacted by their capability, opportunity, and motivation (using the COM-B model) [27] and consider what may support them to take part in the intervention. Strategies will be agreed between the participant and the facilitator and reviewed at each session. The first week’s workbook and session focus on supporting a shared understanding of the structure of the sessions, the expectations for home practice, and the topics planned ahead. For the facilitators, their engagement and adherence to the therapeutic model are supported by a therapist manual which provides steps of what to cover in each session, an adherence checklist and supervision log to complete each week, and weekly supervision with a clinical psychologist. The intervention includes a weekly review process of adherence to home practice by considering factors which may hinder or support adherence which arose in the previous week at the start of the session, as well as for the week ahead at the session's end.

The intervention will be provided by a team of three or four mental health practitioners (e.g., nurse or assistant/trainee psychologists). They will receive weekly supervision and three days’ training in the intervention by a clinical psychologist. The team will also receive monthly supervision by the first author (a senior clinical psychologist with expertise in HD).

### Design and setting

**Fig. 1:**
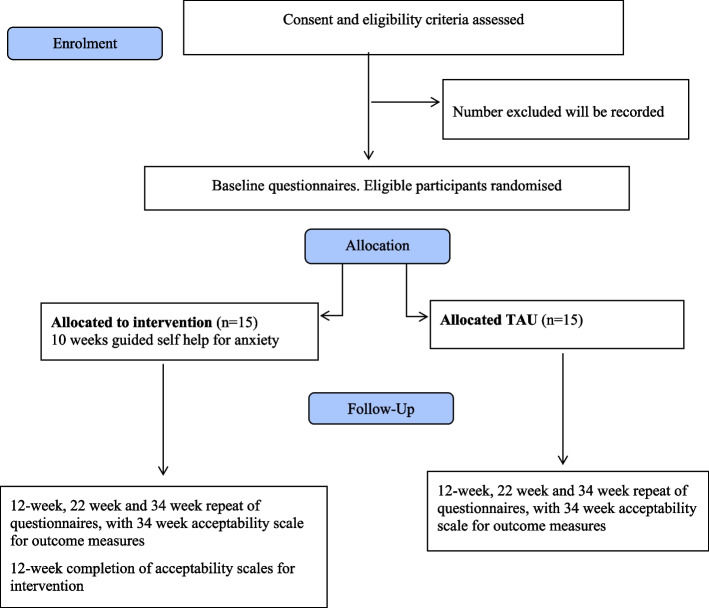
GUIDE-HD study flow diagram using the CONSORT framework (2010)

The design is a 2-arm randomised controlled feasibility trial, collecting both quantitative and qualitative data. The study will compare guided self-help with treatment as usual (TAU), with an allocation ratio of 1:1 to each arm. All participants will receive treatment as usual and not be suspended from any interventions currently offered or planned. The consort flowchart of this study is shown in Fig. 1.

### Participants, eligibility, recruitment and consent

Participants will primarily be recruited across several counties in the UK (Leicestershire, Nottinghamshire, Northamptonshire, Lincolnshire, and Derbyshire) via either local NHS Trusts or UK HD charities (e.g., the Huntington’s Disease Association).

As this is a feasibility study, a formal power calculation is not appropriate given that the parameters for estimation are unclear due to the lack of previous research in this area, and the aim is primarily to assess feasibility outcomes. Consequently, we have assumed that 12 participants per group will be sufficient to determine reliably the primary feasibility outcomes [28]. The total recruitment target is set at 30 HD participants (15 per arm) to allow for expected attrition rates. With the inclusion of carers, the sample size is aimed at 36.

### Eligibility

#### Participant inclusion criteria

Huntington’s disease participants.

- 1. Confirmed genetic test for HD (CAG ≥ 36).
- 2. Diagnosed with clinical anxiety using the Structured Clinical Interview for DSM-5 (SCID-5).
- 3. Premanifest or manifest HD.
- 4. For those who are manifest HD, will be early-stage HD as defined as those with a United Huntington’s Disease Rating Scale (UHDRS) [29] Total Functional Capacity (TFC) score of 9–13.
- 4. If taking a medication known to impact on anxiety, must be stabilised for four weeks.
- 5. Able to read/understand English.
- 6. Age ≥ 18 years.
- 7. Able and willing to give informed consent.

HD carers.

- 1. Age ≥ 18 years.
- 2. Had some involvement in the intervention.
- 3. Able to give informed consent.

#### Participant exclusion criteria

HD participants.

- 1. Current suicidal intent.
- 2. Significant cognitive or communication impairment as determined by the cognitive assessment and clinical psychologist's opinion.
- 3. Unstable medical condition/s.
- 4. Currently receiving another psychological intervention aimed at reducing anxiety.
- 5. Acute psychosis or other acute mental health presentation requiring intense/urgent input from mental health professionals.

**Fig. 2:**
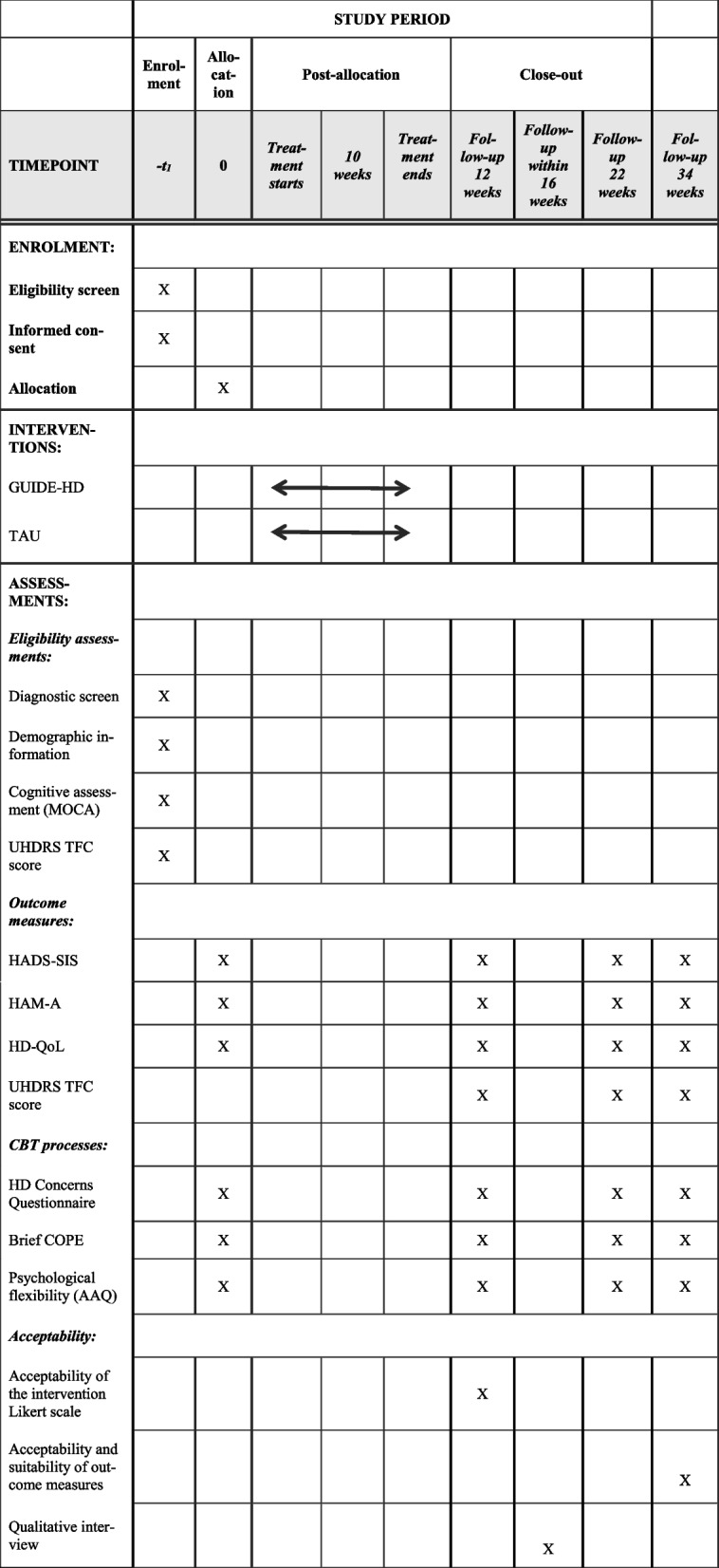
GUIDE-HD schedule of enrolment, interventions, and assessments for participants with the expanded HD gene using the SPIRIT 2013 Statement

Figure 2 shows the data collection timetable.

### Procedure

For recruitment through the NHS, relevant clinical staff will identify any potential participants who appear to meet the inclusion criteria. For participants who express an interest, clinical staff will give a letter of introduction, a participant information sheet, and a “consent-to-contact” form with a stamped addressed envelope. Alternatively, potential participants will be given the option to contact the research team directly or will give verbal consent for the clinical staff member to pass on their contact details to the research team. For participants recruited via charities, the research will be advertised through websites and social media announcements. On this information, contact details for the research team will be given.

On contact with the research team, the participant will be given a further verbal description of the study and have any questions answered. If the participant agrees to go ahead, a face-to-face appointment to take written consent will then be arranged. As part of the consent process, participants will be informed that they are free to discontinue their involvement in the study at any point. Currently, the authors are also pursuing the option of adding remote verbal consent to the study by utilising videoconferencing recording, which will be subject to agreement by the NHS ethics committee. Following consent, potential participants with HD will initially be checked for basic eligibility criteria. If a person meets those basic criteria, then consent will be taken to proceed to the next step of the project. This will involve a clinical diagnostic interview with a clinical psychologist which will be undertaken at a time convenient for the participants, to ascertain whether they meet the *Diagnostic and Statistical Manual of Mental Disorders* (DSM-V; [30]) criteria for an anxiety disorder; this is needed to establish whether full criteria for eligibility will be met. Clinically relevant information gained from this interview will also form part of the treatment formulation plan for those who are allocated to the intervention arm of the study. At this assessment point, if the threshold for an anxiety disorder is reached, then cognitive functioning will also be assessed using a brief measure (the Montreal Cognitive Assessment, MoCA; [31]). The purpose of this measure is both to characterise the sample and enable therapists to understand any difficulties so the intervention can be tailored appropriately. The clinical psychologist will also make a judgement, based on both the cognitive assessment and the interview, whether the potential participant fits the eligibility for cognitive and communication abilities necessary to proceed with the intervention.

Potential participants will be asked whether they have a friend/carer/family member whom they would like to be involved to support them with the intervention. The identified person will be asked to contact the research team to confirm if they wish to be involved. However, having a carer involved is not a requirement for eligibility for the study. The research associate will then either meet with the HD participants or, depending on any current COVID-19 regulations and participant preference, via videoconferencing to collect baseline data using outcome measures for anxiety, depression, irritability, quality of life, functional capacity, HD-related concerns, psychological flexibility, and coping. Brief demographic and clinical data will also be gathered; this will include information about any past or current psychological therapy and their current medications. If participants prefer to complete the measures themselves (except the Hamilton Anxiety Rating Scale, HAM-A; [32], which is clinician-administered), they will be offered this option; this will be either available online or be posted to them along with a stamped addressed envelope to return the forms.

Participants in the intervention arm will receive the guided self-help in addition to their usual care. The intervention will be monitored through facilitators completing an adherence checklist. Each session has its own adherence checklist relating to key elements of the intervention as detailed in the therapist manual. The facilitator is asked to record each item that was completed in the session and date it. These adherence checklists are then sent to the supervisor for review and submitted to the site file. A supervision record is completed to provide an overview for each session and reflective discussions for facilitators in weekly supervision with a clinical psychologist. This will involve opportunity to plan ahead prospective opportunities, to support future adherence, and to review adherence to the intervention in the previous week.

As a feasibility trial of a psychological intervention using established therapies (i.e., CBT and ACT), the risk of adverse events is considered relatively low. However, at follow-up assessments participants will be asked if there are any changes to their medical history, such as receiving a new psychological interventions, changes to their medication, or any changes to their personal circumstances.

The study will adhere to the NHS Code of Confidentiality and will comply with General Data Protection Regulation (GDPR, 2018). All information provided in the study will be kept confidential. Exceptions to this would be if disclosures involving significant risk of harm to self or others were made, in which case the relevant authorities would be informed. No personal identifiable data will be transferred to other members of the research team. All documents will be stored securely and will only be accessible by the study team and authorised personnel.

#### Data collection and management

We will collect both quantitative and qualitative data as part of this study. In addition to the quantitative data collection, participants will also be invited to participate in a qualitative evaluation, along with any carers who supported them.

When outcome measures are being completed remotely, the senior research associate will deliver the assessments via video call conferencing. These assessments will be firstly completed via paper format and later input by the senior research associate into a database created on Microsoft Access, which will contain all results from all outcome measures taken at all timepoints stated in the protocol.

Each participant will be assigned a study code. We will use this code to enter the data into the Access database.

Once the data collection is complete, the data will be transferred to SPSS for analysis. All databases will be password encrypted and pseudo-anonymised. All participants will be assigned a unique ID number, and a separate document linking ID numbers to their actual identities will also be stored in a locked cabinet in a locked office in the first author’s workplace. Electronic information will be stored securely on NHS computers and will be anonymised with ID numbers held on a separate file to those of the participants’ identity.

The anonymised data will be used for analysis purposes and will be shared securely with other members of the study team and as required by our funders. Qualitative data will be in the form of audio recordings and verbatim-anonymised transcripts.

Anonymised transcripts will refer to speakers as interviewers and participants; documents will be password-protected before sharing among members of the team.

Participants will be allowed to withdraw from the study at the point of consent and during the study recruitment period. However, at the point of data anonymisation and analysis, it will no longer be possible to withdraw participants from the study. Following completion of the study, we will archive the data for the time period of five years. We will also ensure that data are prepared and shared in keeping with the rules and regulations of the Jacques and Gloria Gossweiler Foundation (i.e., the study funder).

#### Retention

Strategies to support retention are focused around consultation during the trial design process, adapting the therapy/process to fit the participants’ requirements, developing staff skillset, and attending to the participant experience throughout the trial. With the explicit purpose of maximising retention, the design of the trial and the associated materials were discussed with the aforementioned HD steering group and designed by HD clinicians; this helped the intervention meets the specific needs of people affected by HD. Examples of this included the provision of multiple formats of participant materials (paper, pdf), a facilitated intervention, removal of travel requirements (i.e., home visits or remote contact), posted materials, additional carer support sessions, and direct contact with the research team through multiple methods of communication. To support retention further, all staff involved in the trial who interact with participants will receive specialist training to supplement any existing clinical experience in supporting people and families affected by HD. A further key part of the retention aspect of the study is attending to participant experiences and needs. Further to the general adaptations of the study and training of staff, each participant has an individual psychological formulation, generated by a clinical psychologist from a clinical interview and cognitive screening. This provides an opportunity to tailor further the trial to suit them, for example through identifying any existing strategies that participants use to support their memory and planning. For participants in the intervention arm, this is further built upon through the use of the COM-B model and by collaboratively problem-solving potential barriers to participating in the intervention.

### Feasibility outcomes

#### Primary outcome measures

Feasibility will be assessed using the following:

- 1. Recruitment rate (% eligible participants who consent) by the end of the study.
- 2. Retention of participants in both arms of the trial (% who remain in the study at 3- and 6-month follow-up).

#### Secondary outcome measures

Further feasibility outcomes are as follows:

- 1. Acceptability of the intervention assessed with Likert ratings 0–5 on dimensions of readability, clarity, effort, enjoyment, concentration, helpfulness, and progress regarding therapy materials at 2-week post-intervention and qualitative interviews within 1-month post-intervention.
- 2. Acceptability and suitability of outcome measures assessed with a Likert scale at 6 months, qualitative interviews, and % willing to complete the measures at each timepoint (i.e., baseline, within two weeks after the intervention, 3- and 6-month follow-ups). The acceptability rating scale for the outcome measures will cover overall acceptability, acceptability of the nature of the questions asked in the measures, the time it took to complete the measures, and the number of times they were asked to complete the measures.
- 3. Adherence to the intervention (% of activities accomplished) by the end of the study. This will be determined from the number of items completed on the adherence checklists completed by the facilitators after each session.

In terms of criteria required for progression to a trial, we will establish the feasibility of our study design and intervention using a red-amber-green light system (or otherwise named stop-amend-go). We will use the percentages gained from recruitment, retention, adherence, and acceptability measures and establish feasibility using the following progression criteria:

Definite go (‘green light’) defined as follows:

-  ≥ 60% of eligible participants consent to feasibility trial.
-  ≥ 70% retention of consented participants in both arms of the trial at 3- and 6-month follow-up.
-  ≥ 90% of at least one primary outcome measure is completed, including 6-month follow-up.
-  > 75% of all activities in intervention will be undertaken.
-  > 75% of participants who complete the intervention will agree that the overall intervention is acceptable.

*Amber — for further discussion and modification* as follows:

- 40–59% of eligible participants consent to feasibility trial.
- 50–69% retention of consented participants in both arms of the trial at 3- and 6-month follow-up.
- 70–89% of at least one primary outcome measure completed.
- 60–75% of all activities in intervention will be undertaken.
- 60–75% of participants who complete the intervention will agree that the overall intervention is acceptable.

Definite stop (‘red light’) defined as follows:

-  < 40% of eligible participants consent to feasibility trial.
-  < 50% retention of consented participants in both arms of the trial at 3- and 6-month follow-up.
-  < 70% of both primary outcome measures are not completed.
-  < 60% of all activities in intervention will be accomplished.
-  < 60% of participants who complete the intervention consider the overall intervention to be acceptable.

#### Outcome measures

All participants will be assessed for anxiety using the HADS-anxiety subscale [33] and Hamilton Anxiety Rating Scale (HAM-A; [32]). Reductions on these measures would be the expected primary outcomes of a full RCT. Prior to randomisation, measures will also be collected on depression (HADS-D; [33]), irritability (Snaith Irritability Scale; [34]), quality of life (HD-QoL; [35]), and functional ability (UHDRS TFC; [29]). The putative process or mechanism measures are the HD Concerns Questionnaire [36], a measure of coping (the brief COPE; [37]), and a psychological flexibility questionnaire (AAQ; [38]).

Participants will be asked to complete the questionnaires at baseline, within two weeks after the intervention has finished and at 3-month and 6-month follow-ups post-intervention.

#### Randomisation and blinding

Following baseline measures being undertaken, the research team at Leicestershire Partnership NHS Trust (LPT, who have responsibility for screening and undertaking eligibility assessments and outcome measures and delivering the intervention) will email members of the research team at Lancaster University to inform them that a new recruit needs to be randomised. The LPT team will inform Lancaster University staff whether the new recruit is premanifest or manifest (i.e., whether or not they report motor symptoms consistent with a clinical diagnosis of HD during the baseline demographic questionnaire, coupled with observations by the researcher of any obvious signs of HD movements). Staff at Lancaster University will then undertake a stratified randomisation process using an automated online randomisation system (www.randomization.com), with participants allocated to either the intervention arm or TAU. The stratification for this randomisation is to ensure that those categorised as “premanifest” or “manifest” HD participants are distributed equally across both groups. Block randomisation will be carried out to ensure the control/intervention groups are relatively balanced, with random block sizes of two, with some four, due to the relatively small sample size. The block randomisation design was chosen so the research team are unaware of the next allocation given that the allocation could be from a block of two or a block of four.

Since the senior research associate is undertaking the post-intervention acceptability ratings and qualitative interviews for the intervention group, as well as the repeated outcome measures, blind assessment will not be possible. The research team at Lancaster University are blinded, at the point of randomisation, to the eligibility and baseline measures results.

### Data analysis

To meet the primary objective of determining feasibility of recruitment and retention, descriptive statistics will be used. For the secondary objective of investigating the acceptability of both the intervention and outcome measures, both quantitative and qualitative analyses will be undertaken. For the quantitative analysis, descriptive statistics will also be used to report on findings from the Likert scales. For the acceptability of the intervention, we will examine the percentage of those who reported each score on a 0–5 Likert scale for readability, clarity, effort, enjoyment, concentration, helpfulness, progress regarding therapy materials, and overall acceptability. Adherence to the intervention will also be analysed through descriptive statistics, examining the percentage of activities required by the intervention that are undertaken during the therapy. For the quantitative analysis for the acceptability and suitability of outcome measures, the percentage of the different score into Microsoft Access, the academic researchers on a 0–5 Likert scale will be analysed, as well as the percentage of those who were willing to complete the outcome measures. The analysis for our final objective — i.e., to assess the feasibility criteria from which to progress to a definitive trial, potential clinical effectiveness, and putative mechanisms — will be quantitative, using Likert-scale data from all the measures described above, but will also be informed by feedback from the qualitative analysis.

Regarding the qualitative data, both HD participants and carers in the intervention arm will be interviewed individually within 1-month post-intervention. This will be to gain information regarding experiences of the intervention across the known key parameters of intervention delivery. Data will be digitally recorded, anonymised, and transcribed. Framework analysis [39] will be used as this provides an efficient, focused approach involving specific questions to discuss pre-identified issues [40]. Computer-assisted qualitative data analysis software (e.g., NVivo) may be used to categorise the data.

Data will be stored in Microsoft Access on a corporate server which is subject to regular back-up routines. In terms of ensuring data quality, as all outcome measures are completed in paper format initially and then inputted into Microsoft Access, the academic researchers, prior to analysis, will cross-check paper versions with electronic data entries for any potential input errors. Moreover, no total scores will be inputted for each measure; instead, Access formulas will be used to calculate these for each measure. This represents a prospective approach to managing missing data. As most of the feasibility data to be collected will be known to the research team (e.g., recruitment and retention), the potential for missing data is limited. However, the number of missing responses to the Likert scale assessments will be noted.

All qualitative data transcripts will be transcribed verbatim by the senior research associate, and each researcher will code them individually. Once initial codes are developed, themes will be established and checked with each researcher to ensure inter-rater reliability. Given this is a small-scale feasibility project, no data monitoring committee is being convened for this trial; however, a Lancaster University academic external to the research team will assess adherence to the agreed data analytic strategy and the robustness of the results.

## Discussion

The protocol has been developed as a result of the lack of evidenced interventions for this client group, the prominence of anxiety in clinical presentations, and the negative effects of anxiety on a range of health outcomes for pwHD. In putting together the proposal, we have attended to the needs and views of people affected by HD on the way information is presented.

A number of aspects of the protocol may need to be revised after this initial study, - for example the pace of the intervention, the format of the manual, or the nature of the interaction with the healthcare professional. However, the key indicators of whether this intervention should and could progress to a full trial -  such as our ability to recruit, to support the intervention in a remote fashion rather than in a more traditional (e.g., individual face to face) format, and to see the intervention completed - are the more substantive issues.

Guided self-help is considered a cost-effective psychological therapy for anxiety as it only involves support from a psychological therapist to ‘guide’ the patient to use a self-help intervention. While we have not included any economic evaluation as part of this protocol, we accept that indicators which could be used to assess the cost-effectiveness of the intervention (e.g., less time out of work and less frequent contact with health care services) would need to be addressed in a further trial. Moreover, while we would argue that the provision of psychological interventions for people with HD is important from a health inequality perspective (see [41, 42]), we also accept the limitations of individually focused interventions. Improving psychological well-being, including reducing anxiety, for this group also involves a more radical approach to conceptualising psychological distress from all perspectives — individual and societal [43].

### Dissemination policy

All participants (including carers) will be offered a copy of an accessible and easy to understand summary of the study results. This will be either emailed or posted to the participants depending on their preference. A summary of the results will also be submitted to the funder and to the largest charity in the UK that supports people affected by HD, the Huntington’s Disease Association (HDA). An online meeting will also be scheduled with people affected by HD, giving the opportunity for 2-way interaction. Academic dissemination will take the form of publication in peer-reviewed papers and presentations at international conferences and in other professional fora.

## Conclusion

Psychological therapy has been recommended as the first treatment to be offered to people experiencing anxiety in early-stage HD. Although psychological therapies are recommended for the treatment of anxiety in the general population [44], no trials for anxiety among people with HD have been conducted. This study will build on this previous limited research by feasibility testing a guided self-help therapy, based on content- and process-based CBTs (GUIDE-HD).

## Data Availability

The datasets used and/or analysed during the current study will be made available from the corresponding author on reasonable request.

## Abbreviations

AAQ: Acceptance and Action Questionnaire
ACT: Acceptance and commitment therapy
CI: Chief investigator
CBT: Cognitive behaviour therapy
DSM: *Diagnostic and Statistical Manual of Mental Disorders*
GDPR: General Data Protection Regulation
HADS-SIS: Hospital Anxiety and Depression Scale-Snaith Irritability Scale
HAM-A: Hamilton Anxiety Rating Scale-Anxiety
HD: Huntington’s disease
HDA: Huntington’s Disease Association
HD-QoL: Huntington’s disease-quality of life
HTT: Huntingtin
LPT: Leicestershire Partnership NHS Trust
MOCA: Montreal Cognitive Assessment
NHS: National Health Service
PPI: Patient and public involvement
RCT: Randomised control trial
REC: Research Ethics Committee
TAU: Treatment as usual
UHDRS TFC: United Huntington’s Disease Rating Scale Total Functional Capacity
UK: United Kingdom
